# Cytotoxicity Evaluation of Various Composite Resin Materials: An In Vitro Study

**DOI:** 10.7759/cureus.56169

**Published:** 2024-03-14

**Authors:** Niharika Bhatia, Navaneethan R.

**Affiliations:** 1 Department of Orthodontics and Dentofacial Orthopedics, Saveetha Dental College and Hospitals, Saveetha Institute of Medical and Technical Sciences, Saveetha University, Chennai, IND

**Keywords:** pre-surgical infant orthopedics, infants, mtt assay, cytotoxicity assays, pre-surgical nasal cartilage molding, nasoalveolar molding (nam), dentistry

## Abstract

Aim

This study aimed to determine and compare the cytotoxicity of light-cured composite resin (Enlight light cure composite (Ormco, Glendora, California, USA)), light-cured acrylic resin (Orthocryl LC (Dentaurum, Ispringen, Germany)), and the self-cure acrylic (DPI RR cold cure acrylic (Dental Products of India, Bombay Burmah Trading Corporation Ltd., Mumbai, India)) material and to determine which component is best to be used for the purpose of nasal stent fabrication in the nasoalveolar molding (NAM) technique for cleft therapy.

Methods

Circular discs made from Enlight light cure composite, Orthocryl LC, and self-cure acrylic were submerged for 24 hours in gingival fibroblast media (three discs of each material) and control medium (three discs of each material) that were both contained in plates. After analyzing the optical densities of the plates, the cytotoxicity of the products was assessed by measuring cell viability using the 3-(4,5-dimethylthiazol-2-yl)-2,5-diphenyltetrazolium bromide (MTT) assay. The compiled data was analyzed using IBM SPSS Statistics for Windows, V. 23.0 (IBM Corp., Armonk, NY). The normality of the data was evaluated using the Shapiro-Wilk test. One-way analysis of variance (ANOVA) and pairwise comparison made with Tukey's honestly significant difference (HSD) post hoc test with a significance level (p) of 0.05 were considered.

Results

The percentage of cell viability was between 80% and 150%. A significant mean difference was noted in the cell viability between the three groups (p=0.009). High mean cell viability was seen in Orthocryl LC. However, there was no significant mean difference between Orthocryl LC and Enlight light cure composite material (p=0.854).

Conclusion

Both Orthocryl LC and Enlight light cure composite materials are less cytotoxic when compared to the self-cure acrylic resin material and can be used to fabricate the nasal stent component for infants with cleft defects, undergoing NAM procedure.

## Introduction

Dental resins based on bisphenol A (BPA) are frequently used in orthodontics, preventive dentistry, and restorative dentistry. Complex polymers that comprise a range of stabilizers, initiators, plasticizers, activators, monomers, and other additives make up the composite resins used in dentistry. The two most common monomers are triethylene glycol dimethacrylate (TEGDMA) and bisphenol A-glycidyl methacrylate (Bis-GMA) [1]. BPA is a raw ingredient needed to make Bis-GMA and is never found in its pure form [2].

Concerns regarding the safety of resin matrix components have been raised in recent years due to the growing amount of polymers in the oral cavity. Because composite resins can release components, it is concerning that they can be harmful, despite their growing popularity [3]. In literature, a study by Ferracane found that 15-50% of the methacrylic groups in the organic matrix remained as free monomers post the polymerization stage [4], out of which potentially harmful substances emitted by restorative and bonding composites are TEGDMA and Bis-GMA [5-7]. The most vulnerable individuals involve newborns, small children, and women who are nursing or pregnant. When these components are released into the surrounding tissue, they may have negative local tissue effects or even systemic effects [8-10].

Composite resins are the preferred material in orthodontics for attaching orthodontic brackets to dental enamel [11]. These orthodontic resin materials can be utilized not only for restorative purposes but also for the treatment of neonates with cleft lip and palate deformities [12]. Due to a number of dental defects, patients with cleft lip and palate need substantial restorative procedures. The complicated phenotype of cleft lip and palate results from the disruption of normal embryonic development during pregnancy [13]. For individuals who have a cleft lip, palate, and alveolus, many of whom display traits of social introversion, facial appearance plays a crucial role in their psychosocial development [14,15]. During the neonatal period, the nasal alar cartilages were molded into their proper form and position using the modified nasoalveolar molding (NAM) technique [16,17]. This was accomplished by attaching acrylic nasal stents to the vestibular shield of an oral molding plate. The procedure utilizes the moldability of juvenile cartilage and its capacity to sustain a permanent shape correction. Nevertheless, the resin acrylic substance utilized as a nasal stent for newborns also included Bis-GMA, or TEGDMA, which has been shown to be hazardous, particularly to young children.

Thus, this study aimed to determine whether a new light-cured acrylic resin material could replace more widely used resin or composites for the purpose of nasal molding in infants with cleft lip and palate by comparing its cytotoxicity and inflammatory tissue reactions to those of the conventionally used composite and acrylic resin materials. The authors claim that no studies that were previously performed compare the three chosen materials for the study and their correlation to be used as a treatment option for cleft infants.

## Materials and methods

Preparation of resin discs

Discs having a diameter of 10 mm and a thickness of 1 mm were made using acrylic resins and dental and orthodontic composites (Table 1).

**Table 1:** The three materials used for the study along with their manufacturer details and composition DPI RR: the trade name of the cold cure acrylic resin material used; Bis-GMA: bisphenol A-glycidyl methacrylate; TEGDMA: triethylene glycol dimethacrylate

Manufacturer	Brand name	Resin matrix
Dentaurum, Ispringen, Germany	Orthocryl™ LC	Methyl methacrylate; methyl 2-methylprop-2-enoate; methyl 2-methylpropenoate
Ormco, Glendora, California, USA	Enlight™ light cure adhesive	Bis-GMA and TEGDMA
Dental Products of India, Bombay Burmah Trading Corporation Ltd., Mumbai, India	DPI™ RR cold cure acrylic material	Methyl methacrylate hydroquinone ethyl glycol tertiary amine

**Figure 1:**
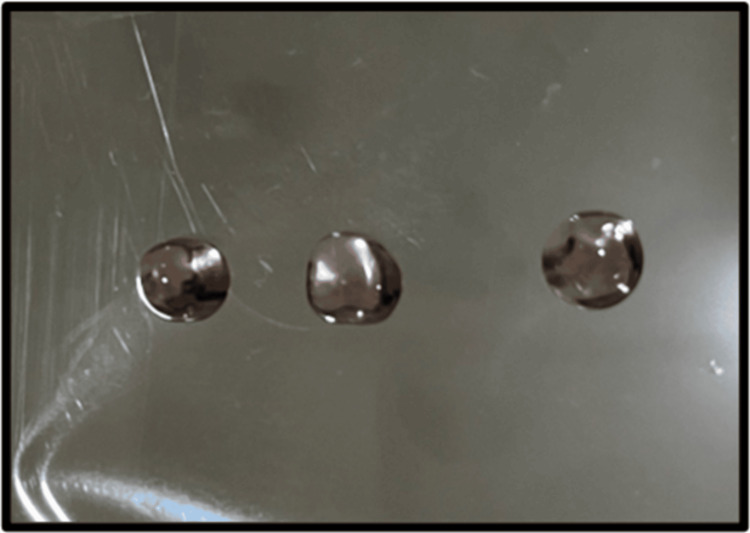
The discs prepared from Orthocryl LC for cytotoxicity testing

Each composite disc was cured on its top surface for 20 seconds each, using the BA Optima 10 LED curing light (BA International Ltd., Northampton, England), which has a wavelength of 420-480 nm and a light intensity of 1,000-1,200 mW/cm^2^ (Figure 1).

Cytotoxicity testing

Standardized cell number of material×(in % of control)=(cell number of material/cell number of glass control)×100% was obtained by calculating the ratio between test and negative control cell counts. When a material's mean standardized cell number is 100%, it can be inferred that the substance is non-toxic because its cell number is the same as that of the glass control.

Dental Pulp Stem Cell (DPSC)

Eagle's Minimal Essential Medium F12 containing 15% (vol/vol) heat-inactivated fetal bovine serum, 50 IU/mL penicillin, 2 mM L-glutamine, and 50 mg/mL streptomycin was used to cultivate DPSC. Incubation conditions were kept as a standard (37°C, 95% air/5% CO2) which were used to grow cells in T-25 cm^2^ culture flasks till confluence (*70%-80%). Following a week, the cells were replated in six-well plates with a cell density of 2.5x10^5^ cells per well after being disintegrated using a trypsin solution. Each well received 2 mL of full Dulbecco's Modified Eagle Medium (DMEM) F12 medium after the cells had been attached for 24 hours.

3-(4,5-Dimethylthiazol-2-yl)-2,5-Diphenyltetrazolium Bromide (MTT) Assay

**Figure 2:**
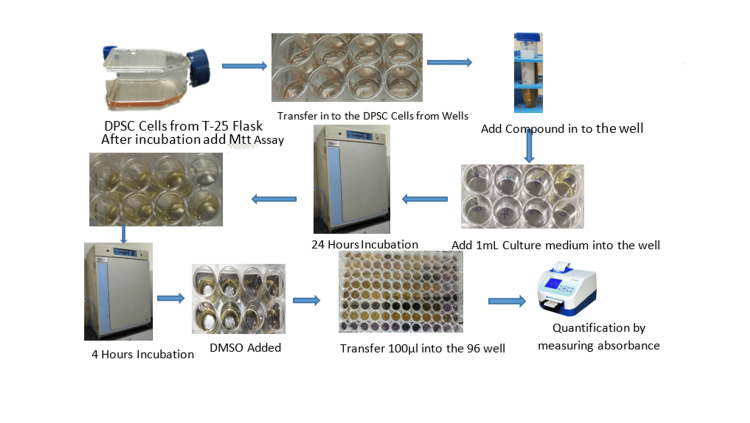
Steps to perform the MTT assay MTT: 3-(4,5-dimethylthiazol-2-yl)-2,5-diphenyltetrazolium bromide; DPSC: dental pulp stem cells; DMSO: dimethyl sulfoxide

One milliliter of full culture media was placed in each well of the six-well plate. Subsequently, the bottom well was filled with 0.5 mg/mL MTT. After that, the plate was incubated for four hours at 37°C. Following the incubation period, the culture media was removed from the insert and well, and 100 µl of dimethyl sulfoxide (DMSO) solution was added to each well to dissolve the formazan crystals that were produced. For two minutes, the cell types were gently shaken to ensure an even mixing of the solvent and blue reaction product. Lastly, for the purpose of measuring cell viability, 100 µl of the colored DMSO was transferred from each insert and well to a fresh 96-well plate. A microplate reader was used to measure the absorbance at 450 nm (Figure 2).

## Results

Statistics

Compiled data was analyzed using IBM SPSS Statistics for Windows, V. 23.0 (IBM Corp., Armonk, NY). The Shapiro-Wilk test was performed for normality testing of the data. The data were found to be normally distributed; hence, the mean comparison of cell viability was elucidated using one-way analysis of variance (ANOVA) (Table 2) and pairwise comparison made with Tukey's honestly significant difference (HSD) post hoc test (Table 3). A significance level of p<0.05 was considered.

**Table 2:** Mean comparison of cell viability of the study groups using the one-way ANOVA test ANOVA: analysis of variance

	Group	Mean±SD	P-value
Cell viability	Self-cure acrylic	0.193±0.024	0.009
	Orthocryl LC	0.321±0.110	
	Enlight light cure adhesive (control)	0.281±0.105	

**Table 3:** Pairwise comparison of cell viability among the study groups

Comparison pairs	Mean difference	P-value	95% confidence interval
Self-cure acrylic vs. Orthocryl LC	-0.163	0.011	-0.293 to -0.034
Self-cure acrylic vs. control	-0.135	0.040	-0.266 to -0.005
Orthocryl LC vs. control	0.028	0.854	-0.101 to 0.158

The results from Table 2 depict that the cell viability between the three groups shows a significant mean difference (p=0.009). High mean cell viability is seen in Orthocryl LC.

**Figure 3:**
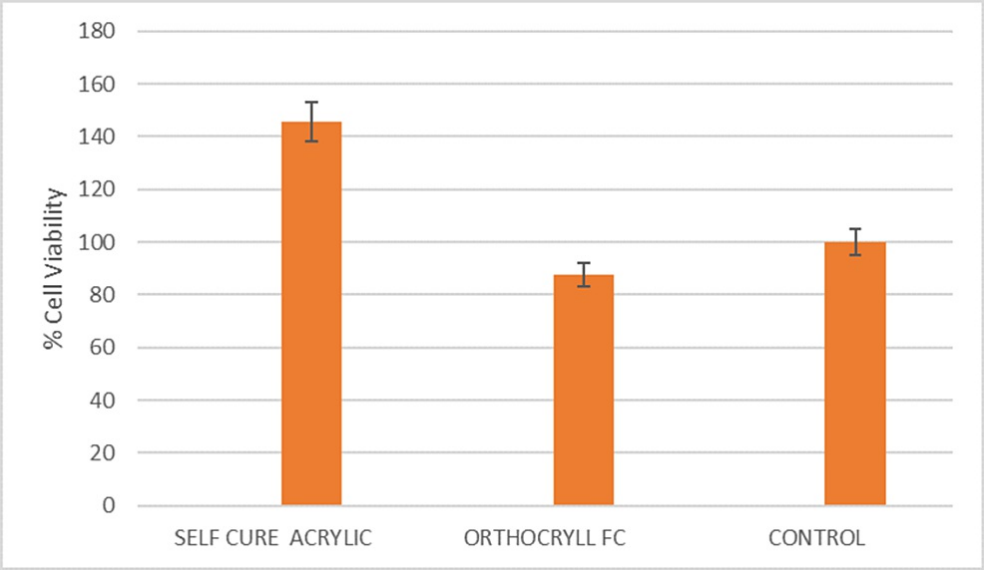
A bar graph of % of cell viability (y-axis) against the material (x-axis) based on the statistics performed

Based on the results of Table 3, there is a significant mean difference in the cell viability between self-cure acrylic and Orthocryl LC (p=0.011) and self-cure acrylic and control (p=0.040). However, the mean difference between Orthocryl LC and the control group is statistically insignificant (p=0.854). These results have been graphically represented in Figure 3 in the form of a bar graph of % of cell viability on the y-axis against the material on the x-axis.

## Discussion

Our study aimed to evaluate the cytotoxicity and tissue response of Orthocryl LC, light-cured composite material, and self-cure acrylic resin material by assessing the gingival fibroblasts' (GFs') cell survival. For this investigation, GFs were selected because, in children with clefts, this tissue is most often in contact with the acrylic and composite material in question.

The ability of cells to continue existing and functioning normally is referred to as cell viability. Given that it indicates the state of health and metabolic activity of cells, it is an essential metric in biological studies. Preserving cell viability is crucial for precise and trustworthy experiment results since changes in viability might impact signaling pathways, biological processes, and overall experiment results. Cell viability is commonly evaluated using the MTT test. In this experiment, mitochondrial dehydrogenases in metabolically active cells convert MTT, a yellow tetrazolium salt, to produce purple formazan crystals. The quantity of viable cells directly correlates with the amount of formazan generated. Thus, cell viability can be measured by researchers by measuring the absorbance of the colored formazan product. The MTT assay is useful for determining how medications, poisons, or other experimental conditions affect the health and proliferation of cells. Because it offers a quantifiable measure of cell viability, it makes it possible to reliably and consistently analyze treatment-induced cytotoxicity or the response of the cell to different stimuli [18].

According to Ferracane [4] and Geurtsen et al. [19], the rate of polymerization can have a substantial impact on the cytotoxicity of composite material. Various monomers found in composite resins, including bisphenol A-glycidyl methacrylate ethoxylated (Bis-EMA), Bis-GMA, TEGDMA, and urethane dimethacrylate (UDMA), have been observed to diffuse from incompletely polymerized materials and demonstrate cytotoxic effects in vitro, as indicated by studies cited by manufacturers [19]. In accordance with many studies, dental adhesives are known to be cytotoxic for GF [20]. Gingival irritation and inflammation are mostly brought on by leftover adhesive monomers.

The results of the current study state that there is a significant mean difference in the cell viability between the three groups of composite/acrylic resin material (p=0.009). High mean cell viability is seen in Orthocryl LC, when compared with the other two groups of composite or acrylic resin material. However, when comparing the cell viability of Orthocryl LC with that of the self-cure acrylic resin material and the control group (light-cured composite material), it is noted that there is a significant mean difference in the cell viability between self-cure acrylic and Orthocryl LC (p=0.011) and self-cure acrylic and control (p=0.040). However, there is no significant mean difference between Orthocryl LC and control (p=0.854). The results of the current study are in accordance with the results of the study performed by Campaner et al. [21] who studied the cytotoxicity of different types of materials on GFs. The study concluded that the cytotoxicity of the self-cure acrylic was greater than the light-cured acrylic materials. Another similar study conducted by Retamoso et al. [22] compared the cytotoxicity of different colored acrylic materials using the MTT assay and concluded that there was statistically no significant difference in the level of cytotoxicity and cell viability between the different groups of acrylic resins. Both these studies however did not compare the three materials, self-cure acrylic resin, Orthocryl LC, and light-cured composite, which have been used in the present study.

However, as this study is in vitro, it has certain limitations. GFs have been employed for the MTT assay, even though the goal was to determine which material could be demonstrated to be the least cytotoxic to be used in the fabrication of the nasal stent component for the NAM procedure. When these materials come into contact with the nasal mucosa, their reactions might change somewhat or not at all.

Limitations

This being an in vitro study has its limitations of the results being questionable when used in vivo. Although cytotoxicity testing has been done on GFs, it has not been performed on nasal mucosa which is imperative as the study aims to evaluate the three composites for their use as nasal stent fabrication for the NAM device. Thus, similar studies can be performed in the future on nasal mucosa tissue for more accurate results.

## Conclusions

When comparing the three different types of materials, self-cure acrylic, Orthocryl LC, and light-cured composite material, that can be used to fabricate a nasal stent component for the technique of NAM in infants with cleft lip and palate defects, it can be concluded that Orthocryl LC has the maximum cell viability and is the least cytotoxic. However, its cytotoxicity is comparable to that of light-cured composite material. The self-cure acrylic material however shows the highest cytotoxicity and should be avoided as a material to be used to fabricate the nasal stent component.
